# The Active for Life Year 5 (AFLY5) school-based cluster randomised controlled trial: effect on potential mediators

**DOI:** 10.1186/s12889-016-2734-5

**Published:** 2016-01-22

**Authors:** Debbie A. Lawlor, Laura D. Howe, Emma L. Anderson, Ruth R. Kipping, Rona Campbell, Sian Wells, Catherine R. Chittleborough, Tim J. Peters, Russell Jago

**Affiliations:** 1 School of Social & Community Medicine, University of Bristol, 39 Whatley Road, Bristol, BS8 2PS UK; 2 MRC Integrative Epidemiology Unit at the University of Bristol, Oakfield House, Oakfield Grove, Bristol, BS8 2BN UK; 3 Centre for Exercise, Nutrition & Health Sciences, School for Policy Studies, University of Bristol, 8 Priory Road, Bristol, BS8 1TZ UK; 4 School of Public Health, University of Adelaide, 178 North Terrace, Adelaide, 5005 South Australia; 5 School of Clinical Sciences, University of Bristol, 69 St Michael’s Hill, Bristol, BS2 8DZ UK

## Abstract

**Background:**

Active for life year 5 (AFLY5) is a school-based intervention, based on social cognitive theory, which aims to promote healthy levels of physical activity and healthy eating by improving a child’s self-efficacy to make healthy choices, their knowledge of how to make such choices and prompting parents to support their children to make healthy choices. Previously published results showed no effect on the three primary outcomes and beneficial effects on three of nine secondary outcomes (time spent screen-viewing at weekends, consumption of snacks and of high energy drinks). This paper aims to determine the effect of the intervention on potential mediators.

**Methods:**

We conducted a cluster RCT of a school-based intervention, with allocation concealed by use of a remote system. The study was undertaken in the South West of England between 2011 and 2013. Participants were school children who were age 8–9 years at baseline assessment and 9–10 years during the intervention. Potential mediators were assessed at the end of the intervention. The intervention consisted of teacher training, provision of all materials required for lessons and homeworks and written materials for school newsletters and parents. The ten potential mediators were child-reported self-efficacy for physical activity and fruit and vegetable consumption, perceived parental logistic support and modelling for their child’s physical activity, parental efforts to limit their child’s sedentary behaviour and modelling of healthy fruit and vegetable consumption, together with a knowledge assessment.

**Results:**

We successfully recruited 60 schools with over 2,221 children; valid data for the 10 mediators were available for 87 % to 96 % of participants. Three of the ten potential mediators were greater in the intervention, compared with the control group: fruit and vegetable self-efficacy 2.2 units (95 % CI: 0.7 to 3.8), assessed on a scale 26 to 130; child-reported maternal limitation of sedentary behaviour 0.5 (0.1 to 0.8), scale 4 to 16; and knowledge 0.5 (0.2, 0.7) scale 0 to 9. Reported maternal limitation of sedentary behaviour and the child’s knowledge explained 23 % of the effect of the intervention on reducing time spent on sedentary behaviour at the weekend. There was no effect on other mediators.

**Conclusions:**

Our findings suggest that the effect of the AFLY5 intervention on reducing screen-viewing at weekends was partially mediated by an effect on mothers limiting their child’s time spent sedentary and on increasing the child’s knowledge about healthy behaviour. However, overall our findings suggest that theory driven interventions, like AFLY5, can fail to influence most potential mediators and this may explain the failure of the intervention to improve most primary and secondary outcomes.

**Trial registration:**

Current Controlled Trials ISRCTN50133740. Registered 17/03/2011

**Electronic supplementary material:**

The online version of this article (doi:10.1186/s12889-016-2734-5) contains supplementary material, which is available to authorized users.

## Background

Low levels of physical activity and fruit and vegetable consumption in childhood are associated with adverse health outcomes, including greater adiposity and associated adverse cardiometabolic risk factors, poorer bone mineralisation, behavioural problems, low mood, and poorer academic attainment [1–7]. Whilst the association of objectively assessed sedentary behaviour with adiposity and cardiovascular risk factors in children has been recently questioned [4], for adults the association of objectively measured high levels of sedentary behaviour with adverse health outcomes appears to be robust [8–10]. There is evidence of tracking into adulthood for all three of physical activity, fruit and vegetable consumption and sedentary behaviour, such that children who acquire healthy levels of these behaviours in childhood tend to maintain them into adulthood [11–13].

Since almost all children attend school, school-based interventions have the potential to efficiently change behaviours to be more health promoting. Recent systematic reviews of school-based interventions aimed at increasing physical activity, decreasing sedentary behaviour and improving fruit and vegetable consumption suggest some beneficial effect. The reviews, however, all highlight the general poor quality of included studies and caution that the pooled results might exaggerate the effectiveness of the interventions [14–19]. In light of this evidence we conducted the Active for Life Year 5 (AFLY5) school-based cluster RCT. AFLY5 aimed to increase time spent in moderate or vigorous physical activity (MVPA), reduce sedentary behaviour and increase fruit and vegetable consumption, using a study design that addressed many of the limitations of previous RCTs in this area [20]. The AFLY5 intervention had no effect on any of the three primary outcomes of accelerometer-assessed physical activity or sedentary behaviour or fruit and vegetable consumption, but did have beneficial effects on three of nine secondary outcomes after adjustment for multiple testing. It reduced the child’s self-reported time-spent on screen viewing at the weekend, consumption of snacks and of high energy drinks, but had no effect on reported screen-viewing during weekdays, consumption of high fat food, mean body mass index, mean waist circumference, odds of general overweight or obesity or odds of central overweight or obesity [21].

Increasing our understanding of the mechanisms of behaviour change is essential for designing and delivering more effective interventions and establishing how to disseminate a complex intervention that has been shown to be effective in one setting to another setting [22]. It is equally important to understand why interventions that are based on sound theory turn out not to be effective [23].

Despite the acknowledged importance of a complex intervention on theory driven mediators [22], few RCTs of complex behaviour change interventions in children have reported mediation effects. Thus, a recent systematic review of systematic reviews concluded that in order to find the most effective physical activity interventions in young people, greater effort needed to be committed to studying mediation of the intervention effect and implementation issues [24]. A review of mediation analyses in RCTs of physical activity interventions in children identified 50 RCTs of 42 different interventions, but only 19 of those reported the effect of the intervention on both theory driven mediators and physical activity [25]. The most common theoretic framework for the interventions was social cognitive theory [26]. Of eleven studies that had relevant data, including on mediators and physical activity outcomes, seven reported beneficial effects on physical activity, but none of those explored the extent to which this was mediated by their theory driven mediators [25]. Of interest, the review did not discuss the importance of determining the effect of a theory driven intervention on potential mediators when effects on the outcomes are null. Yet, as noted above it is important to understand why interventions that are based on well-known theory turn out not to be effective [23]. It is only when this is achieved can we begin to access the extent to which there is empirical support for that theory.

The aim of this study was to determine the effect of the AFLY5 intervention on the potential mediators (child’s self-efficacy and knowledge and their report of parental support and modelling) that the AFLY5 intervention theory was designed to influence, in order to understand whether its lack of effect on the primary outcomes was because it did not affect these more proximal mediators and whether the effect it had on three secondary outcomes was mediated by the hypothesised mechanisms.

## Methods

This paper is written in accordance with the *con*solidated *s*tandards *o*f *r*eporting *t*rials (CONSORT) guidance for the reporting of cluster randomised controlled trials [27].

### Study design and participants

Efforts to reduce bias were taken and have been described fully elsewhere [20, 21, 28]. In short, the trial protocol was published prior to recruitment or data collection and an a priori data analysis plan was agreed upon [20, 28]. Changes to the plan were reviewed by and agreed upon by a TSC [29]. The trial was registered prior to recruitment of schools or data collection (http://www.controlled-trials.com/ISRCTN50133740).

Between March and July 2011 all state primary and junior schools with children in aged 8–11 years in the areas covered by Bristol City Council (93 schools) and North Somerset Council (55 schools) were invited to participate. Both of these areas are in the South West of England and include a range of levels of deprivation, as well as urban and rural areas. Special schools (for children whose additional needs cannot be met in a mainstream setting) were excluded because they are unlikely to be teaching the standard national curriculum and the children may not be able to take part in all the measurements. 148 schools were invited and 63 expressed an interest in taking part and 3 schools subsequently withdrew their interest. 60 schools were recruited (46 in Bristol and 14 in North Somerset). Participants were children in Year 4 (age 8–9) at the time of recruitment.

### Ethical approval and consent

We obtained ethical approval from the University of Bristol Faculty of Medicine and Dentistry Committee for Ethics (reference number 101115). Passive (“opt-out”) parental consent and child assent were obtained. Once schools agreed to participate in the study, parents/guardians of children were sent a letter and information sheet and the opportunity to return an opt-out consent form for each of the measurements. An information sheet for the child was sent at the same time as the letter to the parents. The children were given a second copy of this information sheet at the time that measurements were undertaken and they were asked to give signed assent for each of the measurements.

### Randomisation

Schools were defined as having high or low involvement in any initiatives aimed at increasing physical activity, reducing sedentary behaviour or increasing fruit and vegetable consumption, based on their report of involvement in local or national initiatives, and also by thirds of their score on the English Index of Multiple Deprivation 2010 (IMD 2010) [30]. Schools were grouped into six mutually exclusive strata by these two characteristics and randomly allocated to control or intervention within these strata [20, 28]. Randomisation was undertaken by DAL who was unaware of any characteristics of the schools; it was concealed by using the Bristol Randomised Trials Collaboration’s automated (remote) system.

### Intervention

The intervention was adapted from a previously evaluated US intervention [31], and we had previously tested the feasibility of adapting it for use in the UK and undertaken a pilot RCT [32]. Full details of it have been published [20, 21]. In brief it comprised:

1. Training for Year 5 classroom teachers and learning support assistants, provided by the trial manager, a nutritionist and physical education specialist.
2. Provision of 16 lesson-plans and teaching materials, including pictures, CDs and journals.
3. Provision of 10 parental-child interaction homework activities.
4. Information for schools to insert (as they wished) in the school newsletters about the importance of increasing physical activity, reducing sedentary behaviour and improving diet.
5. Written information for parents on how to encourage their children to eat healthily and be active.

The AFLY5 intervention is based on social cognitive theory [26], and we hypothesised that it would result in beneficial behaviour change by increasing the child’s self-efficacy and knowledge in relation to healthy levels of activity and fruit and vegetable consumption and also by increasing parental support for the child to make healthy choices in relation to these behaviours [20, 28]. Several lessons and homeworks aimed to improve self-efficacy. For example, the ‘*Freeze my TV*’ lesson aimed to provide children with the self-efficacy to replace one period a week when they would usually watch TV with a fun alternative involving some activity with friends or family and the several homeworks and class lessons provided ways of making enjoyable foods (such as pizza and burgers) more healthy. Whilst lessons also provided knowledge about ‘hidden’ unhealthy parts of food, such as how some apparently healthy sounding drinks had substantial added sugar and how unhealthy foods might be promoted specifically to children. The latter was aimed at providing them with knowledge that could increase self-efficacy in relation to not being enticed by such promotions.

The intervention took place when the children were in the school that corresponded to them being age 9–10 years [32]. Schools randomised to the control group continued standard education provision for the school year with no access to the intervention teacher training and no known access to the teaching materials, which had not been published and were not made available by the research team beyond the intervention schools.

### Participant assessments

Baseline assessment (prior to intervention) was undertaken either between April and June 2011 or between September and November 2011, when the children were aged 8 to 9 years (i.e. before and after the school summer break). Follow-up assessment was completed immediately post intervention approximately 12-months after the baseline assessment between April and June 2012 and September and November 2012. Every attempt was made to undertake the assessments in the same order so that the seasons would be similar at both times and a similar difference in age between baseline and outcome assessment would be attained for the children across the schools. Whilst the order was not identical at both time points it was sufficiently similar that the seasons were the same and children assessed prior to the summer break at baseline were also assessed prior to this break at follow-up; with the same true for those assessed after the summer break. With the exception of the knowledge assessment all of the mediating outcomes that are explored in this paper were assessed at both baseline and at outcome assessment; the knowledge test was only performed at the follow-up assessment. Identical protocols and procedures were used at both assessments.

All of the potential mediators were assessed by questionnaires that were combined together into one document and administered in the classroom for the children to complete in the presence of at least one of the trained study fieldworkers who answered any queries and assisted the children with reading and writing according to the study protocol. This instructed them to help with reading and spelling specific words, or understanding the meaning of a particular question and not to suggest answers to any questions. The study fieldworkers had all completed enhanced criminal records bureau (CRB) checks and were blinded to the allocation of schools to the arms of the trial.

Table 1 summarises the ten potential mediators that we assessed. Physical activity self-efficacy was assessed using a validated questionnaire that consists of 26 items, each of which was answered by the child indicating their level of agreement on a 5 point scale (scored 1 to 5), where lower scores on this scale indicated lower self-efficacy [33, 34]. Fruit and vegetable self-efficacy was assessed using a validated questionnaire consisting of 21 items, each of which was answered by the child indicating their level of agreement on a 5 point scale (scored 1 to 5), where lower scores indicate lower self-efficacy [35]. Parental support for physical activity and reducing sedentary behaviour was assessed using a validated 24 item scale, which provides information on modelling of parental physical activity behaviours (5-items for each parent separately), logistical support (3-items for each parent separately) and parental support for reduction of screen viewing (4-items for each parent separately) [36, 37]. Each question is scored between 1–4, with lower scores indicating low levels of modelling or support for physical activity and low levels of limiting sedentary behaviour. Parental modelling of fruit and vegetable consumption was tested using a 12-item validated questionnaire which asked questions about both parents (or care-givers) together [38]. We were unable to identify a validated questionnaire for parental logistic support of fruit and vegetable consumption at the time of starting the study. We developed an assessment to specifically evaluate the knowledge that children in the intervention schools should have gained via the intervention. We piloted the test on children of the same age as participants in AFLY5, but who had no involvement with the study or participants who were in it, in order to make sure that it was understandable to the age of children in AFLY5. The knowledge assessment is shown in Additional file 1: Appendix. It included 9 questions each with three possible responses; the children could score between 0 and 9 on this test.

**Table 1:** Summary of assessments of AFLY5 mediators

Mediator	Range of possible values (number items)^a^	% of children with no missing items in intervention arm (total *n* = 1064)	% of children with no missing items in control arm (total *n* = 1157)	References
Self-reported (validated questionnaire) physical activity self-efficacy	26–130 (26)	B: 96 %	B: 94 %	[33, 34]
		FU: 96 %	FU: 94 %	
Self-reported (validated questionnaire) fruit and veg consumption self-efficacy	21–105 (21)	B: 95 %	B: 93 %	[35]
		FU: 96 %	FU: 94 %	
Child-reported (validated questionnaire) perceived maternal logistic support for physical activity	3–12 (3)	B: 93 %	B: 92 %	[36, 37]
		FU: 95 %	FU: 93 %	
Child-reported (validated questionnaire) perceived paternal logistic support for physical activity	3–12 (3)	B: 88 %	B: 87 %	[36, 37]
		FU: 92 %	FU: 89 %	
Child-reported (validated questionnaire) perceived maternal modelling of physical activity	5–20 (5)	B: 93 %	B: 92 %	[36, 37]
		FU: 95 %	FU: 93 %	
Child-reported (validated questionnaire) perceived paternal modelling of physical activity	5–20 (5)	B: 88 %	B: 87 %	[36, 37]
		FU: 92 %	FU: 89 %	
Child-reported (validated questionnaire) perceived maternal limitation of sedentary behaviour^b^	4–16 (4)	B: 93 %	B: 92 %	[36, 37]
		FU: 93 %	FU: 92 %	
Child-reported (validated questionnaire) perceived paternal limitation of sedentary behaviour^b^	4–16 (4)	B: 87 %	B: 87 %	[36, 37]
		FU: 92 %	FU: 89 %	
Child-reported (validated questionnaire) perceived parental modelling for healthy eating fruit & vegetable consumption^c^	12–48 (12)	B: 95 %	B: 93 %	[38]
		FU: 96 %	FU: 94 %	
Child’s knowledge assessment related to intervention	0–9 (9)	B: N/A	B: N/A	N/A
		FU: 96 %	FU: 94 %	

*B* % with no missing items at baseline, *FU* % with no missing items at follow-up, *N/A* not applicable. We developed the knowledge assessment and it is shown as Additional file 1: Appendix

^a^All variables were treated as continuous variables as detailed in the prior analysis plan [28]

^b^For sedentary behaviour we are not aware of any validated questionnaire assessing parental modelling of healthy sedentary behaviour for use in children, and so have only collected information regarding maternal and paternal limiting of sedentary behaviour for which we were able to identify validated questionnaires

^c^For fruit and vegetable consumption at the time of preparing all data collection tools, we were not aware of any validated questionnaires that provided relevant information for mothers and fathers separately or for logistical support of healthy fruit and vegetable consumption for use in children. We used a questionnaire that had been validated that asked children about parental (either parent or care-giver) modelling for fruit and vegetable consumption

### Statistical analyses

Full details of the analysis plan have been published [28]. We used the approach of Baron and Kenny to examine mediation [39]. As noted in the introduction the main effect of the intervention on outcomes has been published [21]. Here, we first examined the effect of the intervention on potential mediators. This is not only a key stage in Baron and Kenny’s approach, but is of itself valuable even if the intervention has no impact on any of the outcomes. We found that the intervention influenced three of the ten mediators that we assessed (child-report of their self-efficacy for consuming fruit and vegetables and of maternal limitation of their time spent in sedentary behaviour; see full results below). Since the intervention did not affect fruit and vegetable consumption a change in self-efficacy for that could not mediate the (null) outcome effect. Maternal limitation of sedentary behaviour could potentially mediate the effect we had seen on screen-viewing at weekends and the intervention effect on child’s knowledge of healthy eating and activity could mediate any of the three outcomes that the intervention affected. We examined the extent to which the effects of the intervention on the three secondary outcomes were influenced by potential mediators by comparing the standard covariable adjusted effect to one where we additionally adjusted for maternal limiting of sedentary behaviour and knowledge (for the effect on screen viewing at weekends) and additionally adjusted for knowledge (consumption of snacks and high energy drinks).

Multilevel linear regression models were used in order to account for the clustering (non-independence) of children within schools [28]. All analyses included adjustment for the following baseline covariables: age, gender, the baseline measure of the mediator being analysed (except for the knowledge questionnaire which was not assessed at baseline) and the two stratifying variables (school involvement in other health promoting activities and school level deprivation). We used intention to treat (ITT) analyses for our main analyses of the effect of intervention on mediators, with missing data at baseline or follow-up dealt with according to methods suggested by White et al. [40–42]. In these analyses participants were included for each mediator or outcome if they had a follow-up measurement of that mediator or outcome; for missing baseline data we used an indicator variable as described by White and Thompson [42], thus, for each mediator/outcome participants are included even if they do not have a baseline measurement [28]. Note, as there were no measurements of knowledge at baseline, neither it nor an indicator for missing data were included for baseline knowledge.

#### Dealing with item non-response for each of the potential mediators

Irrespective of the number of items each child completed for a given mediator, they were assigned a score that was calculated as the sum of all observed scores plus the sum of missing scores with missing scores replaced with the mean of observed scores [28]. So for example, for a child who had completed 22 items out of the 26 for physical activity efficacy and had a sum of these 22 completed items of 78, the final score was 78 + (4 × (78 ÷ 22)) = 81.5. In addition we generated indicator variables for those with high levels of item non-response, defined as 3 or more missing items for physical activity, fruit and vegetable self-efficacy or parental modelling of fruit and vegetable consumption, and 1 or more missing items for the other mediating outcomes, reflecting the differing number of items for each of these outcomes. In the main analyses we included all participants even if they had high levels of non-response. We undertook sensitivity analyses in which those with high non-response for each mediating outcome were removed to test whether results were influenced by item non-response [28].

## Results

**Fig. 1:**
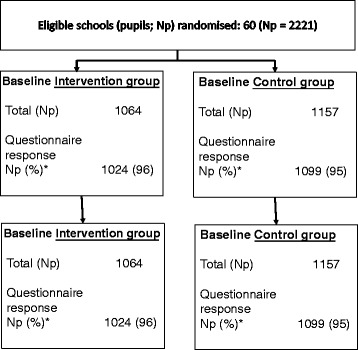
Trial Flow Chart

Figure 1 shows the trial profile. Of the 2,242 potentially eligible students in the 60 participating schools, 10 left the school prior to randomisation and baseline data collection and for 11 their parents or carers did not provide consent to participate in any aspect of the study. All other children (*N* = 2,221; 1064 in the schools that were randomised to intervention and 1157 in the control schools), irrespective of whether they have all measurements or not, are included in the analyses presented here. At each sweep of data collection considerable effort was put into collecting data on those pupils who were absent on the day of data collection for their school, but inevitably some pupils will have been absent on both the main and ‘catch-up’ visits for their school. No child refused assent for completing the questionnaires, thus we have over 87 % with data for each mediator at baseline and over 92 % at follow-up (Table 1). Proportions with data for each measure were similar at both baseline and follow-up and in intervention and control schools.

Baseline characteristics, including mean values for each of the mediator variables were similar amongst pupils randomised to intervention schools and those randomised to control schools (Table 2). Following the intervention, there was evidence that three of the ten potential mediators (fruit and vegetable self-efficacy, maternal limitation of sedentary behaviour and knowledge) were greater in the intervention group compared with the control group (Table 3).

**Table 2:** Comparison of baseline characteristics by randomised group

Characteristic	Unit and type of summary measure	Intervention schools, *N* = 1064		Control schools, *N* = 1157	
		Number	Mean (SD) or N (%)	Number	Mean (SD) or N (%)
Age	Mean (SD) years	1024	9.5 (0.3)	1099	9.5 (0.3)
Physical activity self-efficacy	Mean (SD)	1017	96.0 (15.3)	1085	95.3 (16.0)
Fruit & vegetable self-efficacy	Mean (SD)	1016	87.5 (15.4)	1079	85.7 (17.7)
Perceived maternal logistic support for physical activity	Mean (SD)	989	9.2 (2.4)	1065	9.1 (2.4)
Perceived paternal logistic support for physical activity	Mean (SD)	931	9.0 (2.4)	1002	8.8 (2.6)
Perceived maternal modelling of physical activity	Mean (SD)	991	14.3 (3.8)	1069	14.3 (4.1)
Perceived paternal modelling of physical activity	Mean (SD)	934	15.2 (3.7)	1010	15.0 (3.9)
Perceived maternal limitation of sedentary behaviour	Mean (SD)	989	11.3 (3.5)	1067	11.0 (3.6)
Perceived paternal limitation of sedentary behaviour	Mean (SD)	930	10.8 (3.5)	1003	10.4 (3.6)
Perceived parental modelling of fruit and vegetable consumption	Mean (SD)	1013	33.5 (8.0)	1078	33.0 (8.5)
Categorical variables					
Gender	N (%) female	520	49 %	608	52 %
School participates in other health promoting activity	N (%) yes	800	75 %	711	61 %
School deprivation score	N (%) low	315	30 %	460	40 %
	N (%) medium	368	35 %	345	30 %
	N (%) high	381	36 %	352	30 %
Physical activity self-efficacy	N (%) high level item non-response	13	1 %	13	1 %
Fruit & vegetable self-efficacy	N (%) high level item non-response	9	1 %	12	1 %
Perceived maternal logistic support for physical activity	N (%) high level item non-response	58	5 %	65	6 %
Perceived paternal logistic support for physical activity	N (%) high level item non-response	99	9 %	113	10 %
Perceived maternal modelling of physical activity	N (%) high level item non-response	74	7 %	75	6 %
Perceived paternal modelling of physical activity	N (%) high level item non-response	117	11 %	132	11 %
Perceived maternal limitation of sedentary behaviour	N (%) high level item non-response	58	5 %	59	5 %
Perceived paternal limitation of sedentary behaviour	N (%) high level item non-response	107	10 %	118	10 %
Perceived parental modelling of fruit and vegetable consumption	N (%) high level item non-response	11	1 %	20	2 %

Note some % within categories do not sum to exactly 100 because of rounding

**Table 3:** Main intention to treat analyses of the effect of AFLY5 intervention on potential mediators assessed immediately after the end of the intervention

Outcome	Control group (reference group)		Intervention group		Main effect (group difference)		
	Number	Mean (SD)	Number	Mean (SD)	Number	Difference in means (95 % CI)	*P*-value
Physical activity self-efficacy	1092	97.4 (12.2)	1022	97.4 (13.8)	2114	−0.2 (−1.4 to 1.0)	0.74
Fruit & vegetable self-efficacy	1093	87.2 (15.8)	1020	89.7 (14.4)	2113	2.2 (0.7 to 3.8)	0.005
Perceived maternal logistic support for physical activity	1077	9.5 (2.2)	1006	9.5 (2.3)	2083	−0.1 (−0.3 to 0.1)	0.56
Perceived paternal logistic support for physical activity	1033	9.0 (2.4)	977	9.2 (2.4)	2010	0.1 (−0.1 to 0.3)	0.45
Perceived maternal modelling of physical activity	1079	14.8 (3.6)	1006	14.8 (3.7)	2085	0.1 (−0.2 to 0.3)	0.71
Perceived paternal modelling of physical activity	1033	15.3 (3.6)	975	15.5 (3.7)	2008	0.1 (−0.2 to 0.5)	0.48
Perceived maternal limitation of sedentary behaviour	1078	11.3 (3.5)	1006	11.8 (3.4)	2084	0.5 (0.1 to 0.8)	0.01
Perceived paternal limitation of sedentary behaviour	1031	10.6 (3.5)	977	10.9 (3.5)	2008	0.4 (−0.1 to 0.8)	0.09
Perceived parental modelling of fruit and vegetable consumption	1089	33.9 (7.8)	1017	34.4 (7.9)	2106	0.7 (−0.3 to 1.6)	0.17
Knowledge	1092	7.1 (1.4)	1021	7.5 (1.5)	2113	0.5 (0.2 to 0.7)	<0.001

All differences in means with their 95 % CIs have been estimated using a multi-level linear regression model to account for clustering (non-independence) among children from the same school

The following baseline/school stratifying covariables were included: age, gender, the baseline measure of the mediating outcome under consideration, school involvement in other health promoting activities, school area level deprivation

In these analyses participants were included for each outcome if they had a follow-up measurement of that outcome; for missing baseline data we used an indicator variable as describe by White & Thompson [42], which means for each outcome participants are included even if they do not have a baseline measurement

Table 4 shows the main effect of the intervention on the three secondary outcomes found to be affected by the intervention, both before and after adjustment for potential mediators. Adjustment for maternal limitation of sedentary behaviour and child knowledge attenuated the effect of the intervention on time spent screen viewing at the weekend by 23 %. There was no notable change in the effect of the intervention on consumption of snacks or high energy drinks following adjustment for mediators.

**Table 4:** The main effect of the intervention on the three secondary outcomes found to be affected by the intervention, both before and after adjustment for potential mediators

Outcome	Main effect of the intervention on the outcomes (group difference)^a^				Main effect (group difference) of the intervention on the outcomes after adjusting for relevant potential mediators			
	Number	Difference in means (95 % CI)		*P*-value	Number	Difference in means (95 % CI)		*P*-value
Time spent screen viewing (min/day Saturday)	2121	−20.86	(−37.3, −4.42)	0.01	2083	−16.26^b^	(−33.26, 0.74)	0.06
Servings of snacks (number/day)	2121	−0.22	(−0.38, −0.05)	0.01	2112	−0.20^c^	(−0.37, −0.04)	0.02
Servings of high energy drinks (No/day)	2121	−0.26	(−0.43, −0.1)	0.002	2112	−0.26^d^	(−0.43, −0.09)	<0.001

All differences in means with their 95 % CIs have been estimated using a multi-level linear regression model to account for clustering (non-independence) among children from the same school

The following baseline/school stratifying covariables were included: age, gender, the baseline measure of the mediating variable under consideration, school involvement in other health promoting activities, school area level deprivation

In these analyses participants were included for each outcome if they had a follow-up measurement of that outcome; for missing baseline data we used an indicator variable as describe by White & Thompson [42], which means for each outcome participants are included even if they do not have a baseline measurement

^a^Results are taken from the first publication assessing the effect of interventions of the outcomes at the first follow-up [21]

^b^additionally adjusted for maternal limitation of sedentary behaviour and knowledge as potential mediators

^c^additionally adjusted for fruit and vegetable self-efficacy and knowledge as potential mediators

^d^additionally adjusted for fruit and vegetable self-efficacy and knowledge as potential mediators

### Sensitivity analyses

Results were essentially the same after removing participants with high non-response for each mediating outcome (Additional file 1: Tables S1 and S2).

## Discussion

We have previously shown that AFLY5 did not affect accelerometer assessed time spent in MVPA or sedentary behaviour and nor did it affect fruit and vegetable consumption (our primary outcomes) [21]. After adjustment for multiple testing we found that the intervention successfully reduced child-reported time spent screen viewing at the weekend, consumption of snacks and high energy drinks. Our aim here was to add to the paucity of mediation analyses in theory based complex behaviour interventions in children. We wanted to understand whether an intervention based on social cognitive theory had an impact on potential mediators that reflect that theory, irrespective of the fact that we had already shown the intervention failed to affect all of the primary outcomes and most of the secondary outcomes. For the three secondary outcomes that the intervention did affect, we wanted to determine the extent to which this was due to theory based potential mediators. Both of these are important of understanding whether there is empirical support for the theory.

We found an effect of the intervention on three of the ten potential mediators that we were able to assess and that we anticipated would be affected by our social cognitive theory driven intervention. The intervention increased children’s report of their self-efficacy to consume fruit and vegetables, their mother’s efforts to limit the time they spent screen-viewing and the child’s knowledge related to the key messages of increasing physical activity, reducing sedentary behaviour and healthy eating that the intervention targeted. The latter two explained approximately a quarter of the effect of the intervention on screen viewing at the weekend. At the time of undertaking the trial we were unable to identify any validated tools for assessing child self-efficacy for reducing sedentary behaviour and so the parental efforts to limit this behaviour are the only specific mediators that we assessed for it. It is possible that, had we been able to assess child self-efficacy for reducing sedentary behaviour, the intervention might have increased it and that may further explain some of the effect of the intervention on this outcome. That said, we did not find any effect of the intervention on child self-efficacy for physical activity. Despite increasing the child’s perception of the effort that mothers put into limiting their screen-viewing and increasing child’s knowledge, the intervention did not affect accelerometer assessed sedentary behaviour.

However, we acknowledge that accelerometer assessed time spent in sedentary behaviour is not the same as child-reported screen-viewing and so we might not expect the intervention to affect these two in the same way. First, the two assessment methods differ between a child-report (which may be influenced by reporting bias) and an objective movement sensor. Second, screen-viewing is just one component of sedentary behaviour. Some children may spend a lot of time sedentary via other activities such as reading, socialising with friends or family in a non-active way and playing sedentary games. It is possible that there is some reporting error by the children in the intervention schools in relation to their self-report of screen viewing at weekends and maternal limiting of this behaviour due to the intervention raising awareness of the need to modify this behaviour. Though we saw no effect on other self-report behaviours, including one of the primary outcomes, that of fruit and vegetable consumption [21], and as shown here for the majority of the potential mediators there was no effect.

In addition to an effect on maternal limiting of sedentary behaviour and on child’s knowledge, the intervention increased child self-efficacy for fruit and vegetable consumption, though in our main analyses the intervention did not affect self-reported levels of fruit and vegetable consumption. The disparity between this effect on self-efficacy and outcome suggests that for children increasing self-efficacy is insufficient to change their behaviour in relation to fruit and vegetable consumption. Whilst children of this age have some control over what they eat, parents will be responsible for buying food. We were unable to identify any validated tools for assessing the child’s perception of parental support and role-modelling for fruit and vegetable consumption. Furthermore self-efficacy for a behaviour may increase with an intervention, but that is not the same as the child wanting to change the behaviour that the self-efficacy relates to. Self-efficacy for fruit and vegetable consumption was the only validated dietary self-efficacy questionnaire for children of this age that we could identify at the time of this study but, together with knowledge, it did not mediate the effect of the intervention on either consumption of snacks or high energy drinks. So increasing children’s self-efficacy for fruit and vegetable consumption does not appear to be an effective means of altering other dietary changes.

The intervention did not have an impact on self-efficacy for being able to complete healthy levels of physical activity or the child’s perception of maternal or paternal logistic support or role-modelling of physical activity. Thus, it is perhaps not surprising that the intervention did not impact accelerometer assessed time spent in MVPA [21]. Physical activity in particular might require more intensive or different interventions than the AFLY5 intervention, to increase self-efficacy and provide supportive environments to enable both children and their parents to increase their physical activity. In recent decades there has been a notable decline in children actively travelling to school (ie. on foot or by bike), as opposed to being driven [43]. Contemporary children also spend considerably less time in physically active play both in school and outside school than earlier generations, with reductions in the length of school break time, smaller school playgrounds and less curriculum time dedicated to physical activity lessons, contributing to this [44–46]. Whilst evidence from well conducted RCTs shows that school based physical activity, incorporated into the school curriculum, can be effectively increased [14, 24], it is unclear whether this would have lasting health benefit since to some extent this increase in activity is not a voluntary choice by the child.

Interventions that provide a supportive environment both inside and outside of school might be essential to improve childhood levels of physical activity. This would be consistent with the World Health Organisation concept of Health Promoting Schools (HPS), where children not only learn about healthy behaviours but the school environment is also supportive of healthy behaviours, by, for example, providing secure cycle racks, safe routes (car limited or free) to and from school and healthy foods in canteens. A recent systematic review of the effect of schools adopting the HPS framework identified some positive effects but also noted the need for more robust, high quality evaluation research in this area [47]. There is also evidence that food policy, such as increasing taxes on high sugar foods, is effective in changing children’s diets to healthier ones, but the extent to which politicians will engage with these is unclear [48].

### Study strengths and limitations

The main study design was carefully developed to take account of potential sources of bias that are not addressed by other RCTs in this area [21].

At the time of applying for funding for the study we did not anticipate assessing effects of the intervention on potential mediators, as we have done here. However, these are plausible mediators for our intervention and all have been assessed using questionnaires that have been developed and validated for use in children of the age of those in our trial [33–38]. Given that the original study design did not anticipate these analyses we did not take account of these mediators in our sample size calculation. However, our effect estimates are precisely estimated with narrow 95 % confidence intervals, suggesting we have reasonable power to detect effects if they were present. On the advice of our Trial Steering Committee, we did not adjust for multiple testing here, because mediators might have an impact even if they have a relatively large p-value. This can be illustrated with our results. Had we adjusted for multiple testing (for example using a Bonferroni correction giving a p-value equivalent to 0.05 of 0.05 ÷ 10 = 0.005) one of the three mediators that we have concluded were affected by the intervention (the child’s report of their mothers limiting the time they spent screen viewing) would have been deemed to be null. Yet, in the mediation analyses that appeared to be important in explaining the effect of the intervention on lowering screen-viewing at weekends. All of the mediators were reported by the children, thus, in relation to parental mediators our analyses represent the effect of the intervention on the children’s perception of these. Such perceptions might differ from what the parents actually did, but we would argue that for both the extent to which the parents provide logistic support and for the role modelling of healthy behaviours, the child’s perception is important. For example, parents might undertake most of their physical activity during the day when the child is at school, but that is not likely a useful model for the child.

Whilst all of the mediators that we analysed, with the exception of the knowledge test, have been validated in children and are relevant to the social cognitive theory on which our intervention is based, it is not clear what a given magnitude of difference might mean in terms of a likely effect on an outcome. In part that reflects the paucity of good mediation analysis in this field [25]. However, even without a clear understanding of what a particular magnitude of change might mean we are able to determine whether or not the intervention has an effect on any of the mediators, and if it does what proportion of any effect on outcomes is likely to be driven by the effect on that mediator.

## Conclusions

Our findings suggest that the effect of the AFLY5 intervention on reducing screen-viewing at weekends was partially mediated by an effect on mothers limiting their child’s time spent sedentary and on increasing the child’s knowledge about healthy behaviour. However, overall our findings suggest that theory driven interventions, life AFLY5, can fail to influence most potential mediators and this may explain the failure of the intervention to improve most primary and secondary outcomes. Despite the increased use of appropriate theories in the development of complex interventions, few RCTs of such interventions examine the impact on the mediators that these theories would predict to be effected, particularly in RCTs of children [25]. To some extent our research challenges the notion that an intervention developed on the basis of social cognitive theory had an impact on many of the constructs that that theory would suggest it should influence. However, these findings need further replication and we would suggest that any theory based complex behavioural RCT measures appropriate mediators, assesses the impact of the intervention on these mediators (whether or not it is effective on the outcomes) and determines the extent to which mediators influenced by the intervention explain any effect on outcomes.

## Additional file

Additional file 1: Table S1. Main analyses of the effect of AFLY5 intervention on potential mediators assessed immediately after the end of the intervention, compared to a sensitivity analysis excluding those with high levels of missing data. **Table S2.** The main effect of the intervention on the three secondary outcomes found to be affected by the intervention, both before and after adjustment for potential mediators, and after excluding participants with high levels of missing data for potential mediators. **Appendix.** Knowledge assessment devised by study team. (DOCX 114 kb)

## Abbreviations

AFLY5 Active: For Life Year 5
CDs: Compact discs
CI: Confidence interval
CONSORT: CONsolidated standards of reporting trials
CRB: Criminal Records bureau
HPS: Health Promoting School
IMD: Index of Multiple Deprivation
ITT: Intention to treat
MVPA: Moderate and vigorous physical activity
RCT: Randomised controlled trial
TSC: Trial Steering Committee
UK: United Kingdom
US: United States
