# Supplementary material for: The Active for Life Year 5 (AFLY5) school-based cluster randomised controlled trial: effect on potential mediators
Source: BMC Public Health. 2016 Jan 22;16:68. doi: 10.1186/s12889-016-2734-5 (PMC4724071; doi:10.1186/s12889-016-2734-5)
Supplement: Additional file 1: Table S1. — Main analyses of the effect of AFLY5 intervention on potential mediators assessed immediately after the end of the intervention, compared to a sensitivity analysis excluding those with high levels of missing data. Table S2. The main effect of the intervention on the three secondary outcomes found to be affected by the intervention, both before and after adjustment for potential mediators, and after excluding participants with high levels of missing data for potential mediators. Appendix. Knowledge assessment devised by study team. (DOCX 114 kb) [file 12889_2016_2734_MOESM1_ESM.docx]

**Web-based supplementary material for paper:**

**The Active for Life Year 5 (AFLY5) school-based cluster randomised controlled trial: effect on potential mediators.**

Debbie A Lawlor,^1,2^ Laura D Howe,^1,2^ Emma L Anderson,^1,2^ Ruth R Kipping,^1^ Rona Campbell,^1^ Sian Wells,^1^ Catherine R Chittleborough,^4^ Tim J Peters,^5^ Russell Jago.^3^

^1^ School of Social & Community Medicine, University of Bristol, 39 Whatley Road, Bristol, BS8 2PS

^2^ MRC Integrative Epidemiology Unit at the University of Bristol, Oakfield House, Oakfield Grove, Bristol, BS8 2BN

^3^ Centre for Exercise, Nutrition & Health Sciences, School for Policy Studies, University of Bristol, 8 Priory Road, Bristol BS8 1TZ

^4^ School of Population Health, University of Adelaide, 178 North Terrace, Adelaide, South Australia 5005

^5^ School of Clinical Sciences, University of Bristol, 69 St Michael’s Hill, Bristol BS2 8DZ

**Corresponding author:**

Prof DA Lawlor, MRC Integrative Epidemiology Unit at the University of Bristol, Oakfield House, Oakfield Grove, Bristol, BS8 2BN

Email: [d.a.lawlor@bristol.ac.uk](mailto:d.a.lawlor@bristol.ac.uk)

Telephone: +44 (0)117 3310096

**Web-Supplementary Table 1: Main analyses of the effect of AFLY5 intervention on potential mediators assessed immediately after the end of the intervention, compared to a sensitivity analysis excluding those with high levels of missing data**. Numbers vary by mediator as indicated in the table.

| **Potential mediators** | **Main analysis: the effect of the intervention on the potential mediators, including participants with high levels of missing data** | | |  | **Sensitivity analysis: The effect of the intervention on the potential mediators, excluding those with high levels of missing data for potential mediators** | | |
| --- | --- | --- | --- | --- | --- | --- | --- |
|  | **Number** | **Difference in means (95%CI)** | **p-value** |  | **Number** | **Difference in means (95%CI)** | **p-value** |
| Physical activity self-efficacy | 2114 | -0.2 (-1.4 to 1.0) | 0.74 |  | 2095 | -0.2 (-1.44, 1) | 0.73 |
| Fruit & vegetable self-efficacy | 2113 | 2.2 (0.7 to 3.8) | 0.005 |  | 2100 | 2.2 (0.65, 3.8) | 0.01 |
| Maternal logistic support for physical activity | 2083 | -0.1 (-0.3 to 0.1) | 0.56 |  | 1998 | -0.02 (-0.22, 0.17) | 0.82 |
| Paternal logistic support for physical activity | 2010 | 0.1 (-0.1 to 0.3) | 0.45 |  | 1909 | 0.05 (-0.15, 0.25) | 0.63 |
| Maternal modelling of physical activity | 2085 | 0.1 (-0.2 to 0.3) | 0.71 |  | 1972 | (-0.23, 0.33) | 0.71 |
| Paternal modelling of physical activity | 2008 | 0.1 (-0.2 to 0.5) | 0.48 |  | 1872 | 0.09 (-0.28, 0.46) | 0.64 |
| Maternal limitation of sedentary behaviour | 2084 | 0.5 (0.1 to 0.8) | 0.01 |  | 2004 | 0.5 (0.12, 0.84) | 0.01 |
| Paternal limitation of sedentary behaviour | 2008 | 0.4 (-0.1 to 0.8) | 0.09 |  | 1896 | 0.4 (-0.05, 0.82) | 0.08 |
| Parental modelling of fruit and vegetable consumption | 2106 | 0.7 (-0.3 to 1.6) | 0.17 |  | 2090 | 0.6 (-0.33, 1.52) | 0.21 |
| Knowledge | 2113 | 0.5 (0.2 to 0.7) | <0.001 |  | 2049 | 0.4 (0.21, 0.65) | <0.01 |

All differences in means with their 95% CIs have been estimated using a multi-level linear regression model to account for clustering (non-independence) among children from the same school.

The following baseline / school stratifying covariables were included: age, gender, the baseline measure of the mediating outcome under consideration, school involvement in other health promoting behaviours, school area level deprivation.

In these analyses participants were included for each outcome if they had a follow-up measurement of that outcome; for missing baseline data we used an indicator variable as describe by White & Thompson,([38](#_ENREF_38)) which means for each outcome participants are included even if they do not have a baseline measurement.

**Web-Supplementary Table 2: The main effect of the intervention on the three secondary outcomes found to be affected by the intervention, both before and after adjustment for potential mediators, and after excluding participants with high levels of missing data for potential mediators**

| **Outcome** | **Main effect (group difference) of the intervention on the outcomes ^a^** | | | | **Main effect (group difference) of the intervention on the outcomes after adjusting for relevant potential mediators** | | | | **Sensitivity analysis: The main effect (group difference) of the intervention on the outcomes after adjusting for relevant potential mediators, excluding those with high levels of missing data for potential mediators** | | | |
| --- | --- | --- | --- | --- | --- | --- | --- | --- | --- | --- | --- | --- |
|  | **N** | **Difference in means (95%CI)** | | **p-value** | **N** | **Difference in means (95%CI)** | | **p-value** | **N** | **Difference in means (95%CI)** | | **p-value** |
| Time spent screen viewing (min/day Saturday) | 2121 | -20.86 | (-37.3, -4.42) | 0.01 | 2083 | -16.1^b^ | (-33.04, 0.89) | 0.06 | 1947 | -19.01^b^ | (-36.81, -1.21) | 0.04 |
| Servings of snacks (number/day) | 2121 | -0.22 | (-0.38, -0.05) | 0.01 | 2112 | -0.2^c^ | (-0.37, -0.03) | 0.02 | 2037 | -0.19^c^ | (-0.36, -0.02) | 0.03 |
| Servings of high energy drinks (No/day) | 2121 | -0.26 | (-0.43, -0.1) | 0.002 | 2112 | -0.3^d^ | (-0.43, -0.09) | 0.003 | 2037 | -0.27^d^ | (-0.44, -0.09) | <0.01 |

All differences in means with their 95% CIs have been estimated using a multi-level linear regression model to account for clustering (non-independence) among children from the same school.

The following baseline / school stratifying covariables were included: age, gender, the baseline measure of the mediating outcome under consideration, school involvement in other health promoting behaviours, school area level deprivation.

In these analyses participants were included for each outcome if they had a follow-up measurement of that outcome; for missing baseline data we used an indicator variable as describe by White & Thompson,([38](#_ENREF_38)) which means for each outcome participants are included even if they do not have a baseline measurement.

^a^ Results are taken from the first publication assessing the effect of interventions of the outcomes at the first follow-up ([21](#_ENREF_21))

^b^ additionally adjusted for maternal limitation of sedentary behaviour and knowledge as potential mediators

^c^ additionally adjusted for fruit and vegetable self-efficacy and knowledge as potential mediators

^d^ additionally adjusted for fruit and vegetable self-efficacy and knowledge as potential mediators

**WEB-APPENDIX 1: KNOWLEDGE ASSESSMENT DEVISED BY STUDY TEAM**


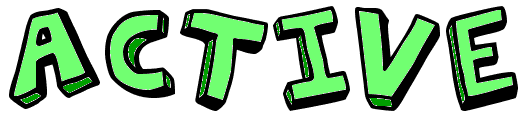

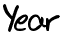

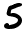

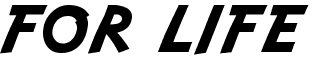


*Office use only – ID label here*

*Office use only – name label here*

**
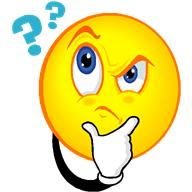
**

**SECTION G**

***Quick Quiz***

**Tick ONE answer for each of the questions below**.

1. How much physical activity do you think children should be doing each day to stay healthy?

| At least 15 minutes | At least 30 minutes | At least 60 minutes |
| --- | --- | --- |
|  |  |  |

1. People who watch TV all day are sometimes called ‘Couch potatoes’! For children to stay healthy, how much time can they spend each day, doing things like watching TV and playing computer games?

| Less than 2 hours | Less than 6 hours | Less than 4 hours |
| --- | --- | --- |
|  |  |  |

1. What do you think the ‘Eatwell Plate’ shows us?

| How to eat lots of food | A healthy balanced diet | What not to eat |
| --- | --- | --- |
|  |  |  |

1. Which of these food groups do you think we should only eat occasionally?

| Bread , rice, potatoes, pasta and other starchy foods | Meat, fish, eggs, beans and other non-dairy sources of protein | Foods and drinks high in fat and/or sugar |
| --- | --- | --- |
|  |  |  |

1. If you were trying to reduce the amount of TV you watched, what would be a healthy alternative?

| Play on a computer | Play outdoors | Surf the internet |
| --- | --- | --- |
|  |  |  |

1. Some snacks are healthy and can be eaten every day, other snacks should only be eaten occasionally (‘sometimes’ snacks). Which of these is an everyday snack?

| Chocolate bar | Crisps | Piece of fruit |
| --- | --- | --- |
|  |  |  |

1. How many portions of fruit or veg do you think you should aim to eat everyday to stay healthy?

| 1 | 5 | 3 |
| --- | --- | --- |
|  |  |  |

1. What is the main reason it is important to eat a healthy balanced breakfast to start the day?

| So you have lots of energy to last until lunchtime | To help you wake up | To keep your parents happy |
| --- | --- | --- |
|  |  |  |

1. Which of these ways of getting to school does **NOT** involve physical activity?

| Cycling | Walking | Travelling by car |
| --- | --- | --- |
|  |  |  |

**
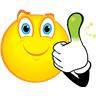
Thank you!**
